# Supplementary material for: Heat adaptation of phage T7 under an extended genetic code
Source: Virus Evol. 2021 Dec 1;7(2):veab100. doi: 10.1093/ve/veab100 (PMC8923235; doi:10.1093/ve/veab100)
Supplement: veab100_Supp [file veab100_supp.zip › Sequencing_Analysis.zip!/Sequencing_Analysis/A3/evidence/MC_SIDE_2_50.html]

BRESEQ :: Evidence


| Predicted mutation | | | | | | |
| --- | --- | --- | --- | --- | --- | --- |
| evidence | seq id | position | mutation | annotation | gene | description |
| MC JC | NC\_001604.1 | 1,253 | Δ1,489 bp | intergenic (–/–) | – / – | –/– |

| Missing coverage evidence... | | | | | | | | | | |
| --- | --- | --- | --- | --- | --- | --- | --- | --- | --- | --- |
|  |  |  | seq id | start | end | size | ←reads | reads→ | gene | description |
| \* | \* | ÷ | NC\_001604.1 | 1253 | 2741 | 1489 | 1819 [50] | [67] 1835 | *–/–* | –/– |

| New junction evidence | | | | | | | | | | | |
| --- | --- | --- | --- | --- | --- | --- | --- | --- | --- | --- | --- |
|  | | seq id | position | reads (cov) | reads (cov) | score | skew | freq | annotation | gene | product |
| \* | ? | NC\_001604.1 | = 1252 | 50 (0.030) | 1744 (1.030) | 316/580 | 0.1 | 96.8% | intergenic (–/–) | *–/–* | –/– |
|  | ? | NC\_001604.1 | 2742 = | 66 (0.040) |  |  |  |  | intergenic (–/–) | *–/–* | –/– |

Only 100 of 1835 total aligned reads displayed.  

`GAGCGTTTCAACAATGATGCCCATTATAAATACGCTGAGATTGCAAGCGACATCATTGATTGCAATTCGGATGAGCATGATGAGTTAACTGGATGGGATGGTGAGTTTGTTGAAACTTGTAAACTAATCCGCAAGTTCTTTGAGGGCATCGCCTCATTCGACATGCATAGCGGGAACATCATGTTCTCAAATGGAGACGTACCATACATCACCGACCCGGTATCATTCTCGCAGAAGAAAGACGGTGGCGCATTCAGCATCGACCCTGAGGAACTCATCAAGGAAGTCGAGGAAGTCGC‑‑ACGACAGAAAGAAATTGACCGCGCTAAGGCCCGTAAAGAACGTCACGAGGGGCGCTTAGAGGCACGCAGATTCAAACGTCGCAACCGCAAGGCACGTAAAGCACACAAAGCTAAGCGCGAAAGAATGCTTGCTGCGTGGCGATGGGCTGAACGTCAAGAACGGCGTAACCATGAGGTAGCTGTAGATGTACTAGGAAGAACCAATAACGCTATGCTCTGGGTCAACATGTTCTCTGGGGACTTTAAGGCGCTTGAGGAACGAATCGCGCTGCACTGGCGTAATGCTGACCGGATGGCT  >  NC_001604.1/2444‑3040`  

                                                                                                                                                                                                                                                                                                          `|`                                                                                                                                                                                                                                                                                                              
`gttttcgaccacgcttacgaaatgctgaaagaaaacatccgttatgatgacatccgtgacactgatgacctgcacgatgctattcacatggctgccgataatgcagttccgcactactacgctgacatctttagcgtaatggcaagtgagggcattgaccttgagttcgaagactctggtctgatgcctgacaccaaggacgtaatccgcatcctgcaagcgcgtatctatgagcaattaacgattgacctctgggaagacgcagaagacttgctcaatgaatacttggaggaagtcgC‑‑Ac                                                                                                                                                                                                                                                                                                          >  1:867‑M2/299‑301 (MQ=255)`  
`gttttcgaccacgcttacgaaatgctgaaagaaaacatccgttatgatgacatccgtgacactgatgacctgcacgatgctattcacatggatgccgataatgcagttccgcactactacgctgacatctttagcgtaatggcaagtgagggcattgaccttgagttcgaagactctggtctgatgcctgacaccaaggacgtaatccgcatcctgcaagcgcgtatctatgagcaattaacgaatgacctcttggaagacgcagaagacttgctcaatgaatacttggaggaagtcgc‑‑ag                                                                                                                                                                                                                                                                                                          >  1:16358‑M2/299‑300 (MQ=255)`  
             `cttacgaaatgctgaaagaaaacatccgttatgatgacatccgtgacactgatgacctgcacgatgctattcacatggctgccgataatgcagttccgcactactacgctgacatctttagcgtaatggcaagtgagggcattgaccttgagttcgaagactccggtctgatgcctgacaccaaggacgtaatccgcatcctgcaagcgcgtatctatgagcaattaacgattgacctctgggaagacgcagaagacttgctcaatgaatacttggaggaagtcgC‑‑ACGACAGAAAGAAAt                                                                                                                                                                                                                                                                                             <  1:144465‑M2/16‑1 (MQ=255)`  
             `attacgaaatgctgaaagaaaaaatccggtatgatgacatccgtgaaactgatgacctgcacgatgcgattcacatggctgccgataatgcagttccgcactactacgctgacatctttagcgtaatggcaagtgagggcattgacattgagttcgaagactctggtctgatgcctgacaccaaggacgtaatccgcatcctgcaagcgcgtatctatgagcaattaacgattgacctctgggaagacgcagaagaattgctcaatgaatacttggaggaagtcgC‑‑ACGACAGAAAGAAAt                                                                                                                                                                                                                                                                                             <  1:342136‑M2/16‑1 (MQ=255)`  
                       `gctgaaagaaaacatccgttatgatgacatccgtgacactgatgacctgcacgatgctattcacatggctgccgataatgcagttccgcactactacgctgacatctttagcgtaatggcaagtgagggcattgaccttgagttcgaagactctggtctgatgcctgacaccaaggacgtaatccgcatcctgcaagcgcgtatctatgagcaattaacgattgacctctgggaagacgcagaagacttgctcaatgaatacttggaggaagtcgC‑‑ACGACAGAAATAAATTGACCGCGCt                                                                                                                                                                                                                                                                                   >  1:104099‑M2/276‑301 (MQ=255)`  
                             `ccgttatgatgacatccgtgacactgatgacctgcacgatgctattcacatggctgccgataatgcagttccgcactactacgctgacatctttagcgtaatggcaagtgagggcatttacctttagttcgaagactctggtctgatgcctgacaccaaggacgtaatccgcatcctgcaagcgcgtatctatgagcaattaacgattgacctctgggaagacgcagaagacttgctcaatgaatacttggaggaagtcgaggaagtcgC‑‑ACGACAGAAAGAAATTGACCGCGCTAAGGcc                                                                                                                                                                                                                                                                             <  1:249581‑M2/32‑1 (MQ=255)`  
                                  `acagccgttatgatgacatccgtgacactgatgacctgcacgatgctattcacatggctgccgataatgcagttccgcactactacgctgacatctttagcgtaatggcaagtgagggcattgaccttgagttcgaagactctggtctgatgcctgacaccaaggacgtaatccgcatcctgcaagcgcgtatctatgagcaattaacgattgacctctgggaagacgcagaagacttgctcaatgaatacttggaggaagtcgC‑‑ACGACAGAAAGAAATTGACCGCGCTAAGGCCCGTaa                                                                                                                                                                                                                                                                        <  1:91815‑M2/37‑1 (MQ=255)`  
                                   `catccgttatgatgacatccgtgacactgatgacctgcacgatgctattcacatggctgccgataatgcagttccgcactactacgctgacatctttagcgtaatggcaagtgagggcattgaccttgagttcgaagactctggtctgatgcctgacaccaaggacgtaatccgcatcctgcaagcgcgtatctatgagcaattaacgattgacctctgggaagacgcagaagacttgctcaatgaatacttggaggaagtcgC‑‑ACGACAGAAAGAAATTGACCGCGCTAAGGCctgtctc                                                                                                                                                                                                                                                                       >  1:285750‑M2/264‑298 (MQ=255)`  
                                   `catccgttatgatgacatccgtgacactgatgacctgcacgatgctattcacatggctgccgataatgcagttacgcactactacgctgacatctttagcgtaatggcaagtgagggcattgaccttgagttcgaagactctggtctgatgcctgacaccaaggacgtaatccgcatcatgcaagcgcgtatctatgagcaattaacgattgacctctgggaagacgcagaagacttgctcaatgaatccttggaggaagtcgC‑‑ACGACAGAAAGAAATTGACCGCGCTAAGGACCGTaaa                                                                                                                                                                                                                                                                       >  1:247333‑M2/264‑301 (MQ=255)`  
                                    `atccgttatgatgacatccgtgacactgatgacctgcacgatgctattcacatggctgccgataatgcagttccgcactactacgctgacatctttagcgtaatggcaagtgagggcattgaccttgagttcgaagactcgggtctgatgcctgacaccaaggacgtaatccgcatcctgcaagcgcgtatctatgagcaattaacgattgacctctgggaagacgcagaagacttgctcaatgaatacttggaggaagtcgC‑‑ACGACAGAAAGAAATTGACCGCGCTAAGGCCCGTAAAg                                                                                                                                                                                                                                                                      <  1:328456‑M2/39‑1 (MQ=255)`  
                                         `catccgtgacactgatgacctgcacgatgctattcacatggctgccgataatgcagttcagcactactacgctgacatctttagcgtaatggcaagtgagggcattgaccttgagttcgaagactctggtctgatgcctgacaccaaggacgtaatccgcatcctgcaagcgcgtatctatgagcaattaacgattgacctctgggaagacgcagaagacttgctcaatgaatacttggtggaagtcgaggaagtcgC‑‑ACGACAGAAAGAAATTGACCGCGCTAAGGCCCgtaaagctgtt                                                                                                                                                                                                                                                                 >  1:273334‑M2/258‑296 (MQ=255)`  
                                             `gatgacatccgtgacactgatgacctgcacgatgctattcacatggctgccgataatgcagttccgcactactacgctgacatcattagcgtaatggcaagtgagggcattgaccttgagttcgaagactctggtctgatgcctgacaccaaggacgtaatccgcatcctgcaagcgcgtatctatgagcaattaacgattgacctctgggaagacgcagaagacttgctcaatgaatacttggaggaagtcgC‑‑ACGACAGAAAGAAATTGACCGCGCTAAGGCCCGTAAAGAACGTCACg                                                                                                                                                                                                                                                             <  1:49026‑M2/48‑1 (MQ=255)`  
                                                  `catccgttacactgatgacctgcacgatgctattcacatggctgccgataatgcagttccgcactactacgctgacatctttagcgtaatggcaagtgagggcattgaccttgagttcgaagactctggtctgatgcctgacacgaaggacgtaatccgcatcctgcaagcgcgtatctatgagcaattaacgattgacctctgggaagacgcagaagacttgctcaatgaatacttggaagaagtcgC‑‑ACGACAGAAAGAAATTGACCGCGCTAAGGCCCGTAAAGAACGTCAcgagctg                                                                                                                                                                                                                                                        >  1:304509‑M2/249‑298 (MQ=255)`  
                                                  `aactgatgacctgcacgatgctattcacatggctgccgataatgcagttccgaactactacgctgacatctttagcgtaatggcaagtgagggcattgaccttgagttcgaagactctggtctgatgcctgacaccaaggacgtaatccgcatcctgcaagcgcgtatctatgagcaattaacgattgacctatgggaagacgcagaagacttgctcaatgaatacttggaggaagtcgaggaagtcgC‑‑ACGACAGAAAGAAATTGACCGCGCTAAGGCCCGTAAAGAACGTCACGAgggg                                                                                                                                                                                                                                                        <  1:29279‑M2/53‑1 (MQ=255)`  
                                                            `aggcacgatgcttttcacatggctgccgataatgcagttccgcactactacgctgacatctttagagtaatggcaagtgaaggcattaaccttaagttcgaagactctggtctgatgcctgaccccaagaacgtaatccgcatcctccaaccgcttatctatgagcaattaacgattaacctctgggaagacgcagaagacttgctcaatgaatacttggatgaagtcgaggaagtcgC‑‑ACGACAGAAAGAAATTGACCGCGCTAAGGCCCGTAAAGAACGTCAAGAGGGGAGCTTAGAgg                                                                                                                                                                                                                                              <  1:326123‑M2/63‑1 (MQ=255)`  
                                                              `gcacgatgctattcacatggcttccgataatgcagttccgcactactacgctgacatctttagcgtaatggcaagtgagggcattgaccttgagttcgaagactctggtctgatgcctgacaccaaggacgtaatccgcatcctgcaagcgcgtatctatgagcaattaacgattgacctctgggaagacgcagaagacttgctcaatgaatacttggaggaagtcgaggaagtcgC‑‑ACGACAGAAAGAAATTGACcgcgctaatgcccgtaaagaacgacctgtctcttatacacatctc                                                                                                                                                                                                                                            >  1:263768‑M2/237‑279 (MQ=255)`  
                                                                   `atgctattcacatggctgccgataatgcagttccgcactactacgctgacatctttagcgtaatggcaagtgagggcattgaccttgagttcgaagactctggtctgatgcctgacaccaaggacgtaatccgcatcctgcaagctcgtatctatgagcaattaacgattgacctctgggaagacgcagaagacttgctcaatgaatacttggaggaagtcgaggaagtcgC‑‑ACGACAGAAAGAAATTGACCGCGCTAAGGCCCGTAAAGAACGTCCCGAGGGGGGATTAGCGGCATGCAg                                                                                                                                                                                                                                       >  1:255682‑M2/232‑301 (MQ=255)`  
                                                                            `tgtataagagacaggctgccgaaaatgcagttccgcactactacgctgacatctttagcgtaatggcaagtgagggcattgaccttgagttcgaagactctggtctgatgcctgacaccaaggacgtaatccgcatcctgcaagcgcgtatctatgagcaattaacgattgacctctgggaagacgcagaagacttgctcaatgaatacttggaggaagtcgC‑‑ACGACAGAAAGAAATTGACCGCGCTAAGGCCCGTAAAGAACGTCACGAGGGGCGCTTAGAGGCACGCAGATTCAAACg                                                                                                                                                                                                                              <  1:322660‑M2/79‑1 (MQ=255)`  
                                                                               `tggctgccgataatgcagttccgcactactacgctgacatctttagcgtaatggcaagtgagggcattgaccttgagttcgaagactctggtctgatgcctgacaccaaggacgtaatccgcatcctgcaagcgcgtatctatgagcaattaacgattgacctctgggaagacgcagaagacttgctcaatgaatacttggaggaagtcgaggaagtcgC‑‑ACGAAAGAAAGAAATTGACCGCGCTAAGGCCCGTAAAGAACGTCACTAGGGGCGCTTAGAGGCACGCAGATTCAAACGTCg                                                                                                                                                                                                                           >  1:27044‑M2/220‑301 (MQ=255)`  
                                                                                   `aatgtgtataagagacaggcactactacgctgacatctttagcgtaatggcaagtgagggcattgaccttgagttcgaagactctggtctgatgcctgacaccaaggacgtaatccgcatcctgcaagcgcgtatctatgagcaattaacgattgacctctgggaagacgcagaagacttgctcaatgaatacttggaggaagtcgaggaagtcgC‑‑ACGACAGAAAGAAATTGACCGCGCTAAGGCCCGTAAAGAACGTCACGAGGGGCGCTTAGAGGCACGCAGATTCAAACGTCGCAAc                                                                                                                                                                                                                       <  1:60351‑M2/86‑1 (MQ=255)`  
                                                                                     `ccgataatgcagttccgcactactacgctgacatctttagcgtaatggcaagtgagggcattgaccttgagttcgaagactctggtctgatgcctgacaccaaggacgtaatccgcatcctgcaagcgcgtatctatgagcaattaacgattgacctctgggaagacgcagaagacttgctcaatgaatacttggaggaagtcgaggaagtcgC‑‑ACGACAGAAAGAAATTGACCGCGCTAAGGCCCGTAAAGAACGTCACGAGGGGCGCTTAGAGGCACGCATATTCAAACGTCGCAACCg                                                                                                                                                                                                                     <  1:39758‑M2/88‑1 (MQ=255)`  
                                                                                      `caataatgcaggtccgcactacgacgcgaacatctttagcgtaatggcaagtgagggcattgaacttaagttcgaagactctggtctgatgcctgacaccaaggacgtaatcctcatcctgcaagcgcgtatctatgagcaattaacgattgacctctgggaagacgcagaagacttgctcaatgaatacttggaggaagtcgaggaagtcgC‑‑ACGACAGAAAGAAATTGACCGCGCTAAGGCCCGTAAAGAACGTCACGAGGGGCGCTTAGAGGCACGCAGATTCAAACGTCGCAACCGc                                                                                                                                                                                                                    <  1:156092‑M2/89‑1 (MQ=255)`  
                                                                                           `gcagaagagacagctaatacgctgacatctttagcgtaagggcaagtgagggcattgaccttgagttcgaagactctggtctgatgcctgacaccaaggacgtaatccgcatcctgcaagcgcgtatctatgagcaattaacgattgacctctgggaagacgcagaagacttgctcaatgaatacttggaggaagtagaggaagtggC‑‑ACGACAGAAAGAAATTGACCGCGCTAAGGCCCGTAAATAACGTCACGAGGTGCGCTTAGAGGCACGCAGATTCAAACGTCGCAACCGCAAGGc                                                                                                                                                                                                               <  1:19555‑M2/94‑1 (MQ=255)`  
                                                                                           `ctgccgataatgcagttccgcactactacgctgacatctttagcgtaatggcaagtgagggcattgaccttgagttcgaagactctggtctgatgcctgacaccaaggacgtaatccgcatcatgcaagcacgtatctatgagcaattaacgattgacctctgggaagacgcagaagacttgctcaatgaatacttggaggaagtcgC‑‑ACGACAGAAAGAAATTGACCGCGCTAAGGCCCGTAAAGAACGTCACGAGGGGCGCTTAGAGGCACGCAGATTCAAACGTCGCAACCGCAAGGc                                                                                                                                                                                                               <  1:189693‑M2/94‑1 (MQ=255)`  
                                                                                                    `atgcagttccgcactactacgctgacatctttagcgtaatggcaagtgagggcattgaccttgagttcgaagactctggtctgatgcctgacaccaaggacgtaatccgcatcctgcaagcgcgtatctatgagcaattaacgattgacctctgggaagacgcagaagacttgctcaatgaatacttggaggaagtcgC‑‑ACGACAGAAAGAAATTGACCGCGCTAAGGCCCGTAAAGAACGTCACGAGGGGCGCTTAGAGGCACGCAGATTCAAACGTCGCAACCGCAAGGCAcgtaactg                                                                                                                                                                                                      >  1:146916‑M2/199‑298 (MQ=255)`  
                                                                                                        `ctactacgctgacatctttagcgtaatggcaagtgagggcattgaccttgagttcgaagactctggtctgatgcctgacaccaaggacgtaatccgcatcccgcaagcgcgtatctatgagcaattaacgattgacctctgggaagacgcagaagacttgctcaatgaatacttggaggaagtcgaggaagtcgC‑‑ACGACAGAAAGAAATTGACCGCGCTAAGGCCCGTAACGAACGTCACGAGGGGCGCTTAGAGGCACGGAGATTCAAACGTCGCAACCGCAA‑GCACGTAAAGcacaca                                                                                                                                                                                                 >  1:19972‑M2/195‑301 (MQ=255)`  
                                                                                                        `ctactacgctgacaactttagcgtaatggcaagtgagggcattgaccttgagttcgaagactctggtctgatgcctgacaccaaggacgtaatccgcatcctgcaagcgcgtatctatgagcaattaacgattgacctctgggaagacgcagaagacttgctcaatgaatacttggaggaagtcgaggaagtcgC‑‑ACGACAGAAAGAAATTGACCGCGCTAAGGCCCGTAAAGAACGTCACGAGGGGCGCTTAGAGGCACGCAGATTCACACGTCGCAACCGCAAAGCACGTAAagcactg                                                                                                                                                                                                  >  1:310193‑M2/195‑299 (MQ=255)`  
                                                                                                        `agttccgcactactacgctgacatctttagcgtaatggcaagtgagggcattgaccttgagttcgaagactctggtctgatgcctgacaccaaggacgtaatccgcatcctgcaagcgcgtatctatgagcaattaacgattgacctctgggaagacgcagaagacttgctcaatgaatacttggaggaagtcgC‑‑ACGACAGAAAGAAATTGACCGCGCTAAGGCCCGTAAAGAACGTCACGAGGGGCGCTTAGAGGCACGCAGATTCAAACGTCGCAACCGCAAGGCACGTAAAGcacac                                                                                                                                                                                                  <  1:124952‑M2/107‑1 (MQ=255)`  
                                                                                                          `ttccgcactactacgcggacatctttagcgtaatggcaagtgagggcatttaccttgagttcgaagactctggtctgatgccagacaccaaggacgtaagccgcatcctgcaagcgcgtatctatgagcaattaacgattgacctctgggaagacgcagaagacttgctcaatgaatacttggaggaagtcgC‑‑ACGACAGAAAGAAATTGACCGCGCTAAGGCCCGTAAAGAACGTCACGAGGGGCGCTTAGAGGCACGCAGATTCAAACGTCGCAACCGCAAGGCACGTAAAGCACACaa                                                                                                                                                                                                <  1:289635‑M2/109‑1 (MQ=255)`  
                                                                                                            `ccgcactactacgctgacctctttagcgtaagggcaagtgaggtcattgaccttgagttcgaagactctggtctgatgcctgacaccaaggacgtaatccgcatcctgcaagcgcgtatctatgagcaattaacgattgacctctgggaagacgcagaagacttgctcaatgaatacttggaggaagtcgC‑‑ACGACAGAAAGAAATTGACCGCGCTAAGGCCCGTAAAGAACGTCACGAGGGGCGCTTAGAGGCACGCAGATTCAAACGTCGCAACCGCAAGGCACGTAAAGCACACAAAg                                                                                                                                                                                              <  1:266483‑M2/111‑1 (MQ=255)`  
                                                                                                            `ccgcactactacgctgacatctttagcgtaatggcaagtgagggcattgaccttgagttcgaagactctggtctgatgcctgacaccaaggacgtaatccgcatcctgcaagcgcgtatctatgagcaattaacgattgacctctgggaagacgcagaagacttgctcaatgaatacttggaggaagtcgC‑‑ACGACAGAAAGAAATTGACCGCGCTAAGGCCCGTAAAGAACGTCACGAGGGGCGCTTAGAGGCACGCAGATTCAAACGTCGCAACCGCAAGGCACGTAAAGCACAcaaaa                                                                                                                                                                                              >  1:46744‑M2/191‑300 (MQ=255)`  
                                                                                                                         `aagagacagtggcaagtgagggcattgaccttgagttcgaagactctggtctgatgcctgacaccaaggacgtaatccgcatcctgcaagcgcgtatctatgagcaattaacgattgacctctgggaagacgcagaagacttgctcaatgaatacttggaggaagtcgaggaagtcgC‑‑ACGACAGAAAGAAATTGACCGCGCTAAGGCCCGTAAAGAACGTCATGAGGGGCGCTTAGATGCACGCAGATTCAAACGTCGCAACCGCAAGGCACGTAAAGCACACAAAGCTAAGCGCGAAAg                                                                                                                                                                                 <  1:9518‑M2/124‑1 (MQ=255)`  
                                                                                                                              `atctttagcgtaatggcaagtgagggcattgaccttgagttcgaagactctggtctgatgcctgacaccaaggacgtaatccgcatcctgcaagcgcgtatctatgagcaattaacgattgacctctgggaagacgcagaagacttgctcaatgaatacttggaggaagtcgC‑‑ACGACAGAAAGAAATTGACCGCGCTAAGGCCCGTAAAGAACGTCACGAGGGGCGCTTAGAGGCACGCAGATTCAAACGTCGCAACCGCAAGGCACGTAAAGCACACAAAGCTAAGCGCGAAAGAAtgc                                                                                                                                                                            <  1:65510‑M2/129‑1 (MQ=255)`  
                                                                                                                                `ctttagcgtaatggcaagtgagggcattgaccttgagttcgaagactctggtctgatgcctgacaccaaggacgtaatccgcatcctgcaagcgcgtatctatgagcaattaacgattgacctctgggaagacgcagaagacttgctcaatgaatacttggaggaagtcgC‑‑ACGACAGAAAGAAATTGACCGCGCTAAGGCCCGTAAAGAACGTCACGAGGGGCGCTTAGAGGCACGCAGATTCAAACGTCGCAACCGCAAGGCCCGTAAAGCACACAAAGCTAAGAGCGAAAGAAtgctt                                                                                                                                                                          >  1:281258‑M2/171‑301 (MQ=255)`  
                                                                                                                                      `gacagtggcaagtgagggcattgaccttgagttcgaagactctggtctgatgcctgacaccaaggacgtaatccgcatcctgcaagcgcgtatctatgagaaattaacgattgacctctgggaagacgcagaagacttgctcaatgaatacttggaggaagtcgC‑‑ACGACAGAAAGAAATTGACCGCACTAAGGCCCGTAAAGAACGTCACGAGGGGCGCTTAGAGGCACGCAGATTCAAACGTCGCAACCGCAAGGCACGTAAAGCACACAAAGCTAAGCGCGAAAGAATGCTTGCTGCg                                                                                                                                                                    <  1:295720‑M2/137‑1 (MQ=255)`  
                                                                                                                                        `gtgagggcatttaccttgagttcgaagactctggtctgatgcctgacaccaaggacgtaatccgcatcctgcaagcgcgtatctatgagcaattaacgattgacctctgggaagacgcagaagacttgctcaatgaatacttggaggaagtcgaggaagtcgC‑‑ACGACAGAAAGAAATTGACCGCGCTAAGGCCCGTAAAGAACGTCACGAGGGGCGCTTAGAGGCACGCAGATTCAAACGTCGCAACCGCAAGGCACGTAAAGCACACAAAGCTAAGAGCGCAAGAATGCTTGATGCGTg                                                                                                                                                                  >  1:109671‑M2/163‑301 (MQ=255)`  
                                                                                                                                                `agtgagggcattgaccttgagttcgaagactctggtctgatgcctgacaccaaggacgtaatccgcatcctgcaagcgcgtatctatgagcaattaacgattgacctctgggaagacgcagaagacttgctcaatgaatacttggaggaagtcgC‑‑ACGACAGAAAGAAATTGACCGCGCTAAGGCCCGTAAAGAACGTCACGAGGGGCGCTTAGAGGCACGCAGATTCAAACGTCGCAACCGCAAGGCACGTAAAGCACACAAAGCTAAGCGCGAAAGAATGCTTGCTGCGTggcgactgt                                                                                                                                                          >  1:275457‑M2/155‑297 (MQ=255)`  
                                                                                                                                                  `tgatggcattgaccttgagttcgaagactctggtctgatgcctgacaccaaggacgtaatccgcatcctgcaagcgcgtatctatgagcaattaacgattgacctctgggaagacgcagaagacttgctcaatgaatacttggaggaagtcgC‑‑ACGACAGAAAGAAATTGACCGCGCTAAGGCCCGTAAAGAACGTCACGAGGGGCGCTTAGAGGCACGCAGATTCAAACGTCGCAACCGCAAGGCACGTAAAGCACACAAAGCTAAGCGCGAAAGAATGCTTGCTGCGTGGCGATGGgcg                                                                                                                                                        >  1:301829‑M2/153‑300 (MQ=255)`  
                                                                                                                                                       `gcattgaccttgagttcgaagactctggtctgatgcctgacaccaaggacgtaatccgcatcctgcaagcgcgtatctatgagcaattaacgattgacctctgggaagacgcagaagacttgctcaatgaatacttggaggaagtcgC‑‑ACGACAGAAAGAAATTGACCGCGCTAAGGCCCGTATAGAACGTCACGAGGGGCGCTTAGAGGCACGCAGATTCAAACGTCGCAACCGCAAGGCACGTAAAGCACACAAAGCTAAGCGCGAAAGAATGCTTGCTGCGTGGCGATGGGCTGAACg                                                                                                                                                   >  1:112957‑M2/148‑301 (MQ=255)`  
                                                                                                                                                           `gttcgaagactctggtctgatgcctgacaccaaggacgtaatccgcatcctgcaagcgcgtatctatgagcaattaacgattgacctctgggaagacgcagaagacttgctcaatgaatacttggaggaagtagaggaagtcgC‑‑ACGATAGAAAGAAATTGACCGCGCTAAGGCCCGTAAAGAACGTCACGAGGGGCTCTTAGAGGCACGCAGATTCAAACGTCGCAACCGCAAGGCACGTAAAGCACACAAAGCTAAGCGCGAAAGAATGCTTGCTGCGTGGCGATGGGCTGAACGTCaa                                                                                                                                               <  1:89749‑M2/158‑1 (MQ=255)`  
                                                                                                                                                            `gaccttgagttcgaagactctggtctgatgcctgacaccaaggacgtaatccgcatcctgcaagcgcgtatctatgagcaattaacgattgacctctgggaagacgcagaagacttgctcaatgaatacttggaggaagtcgC‑‑ACGACAGAAAGAAATTGACCGCGCTAAGGCCCGTAAAGAACGTCACGAGGGGCGCTTAGAGGCACGCAGATTCAAACGTCGCAACCACAAGGCACGTAAAGCACACAAAGCTAAGCGCGAAAGAATGCTTGCTGCGTGGCGATTGGCTGAACGTCAAg                                                                                                                                              >  1:243833‑M2/143‑301 (MQ=255)`  
                                                                                                                                                             `accttgagttcgaagactctggtctgatgcctgacaccaaggacgtaatccgcatcctgcaagcgcgtatctatgagcaattaacgattgacctctgggaagacgcagacgacttgctcaatgaatacttggagtacgtcgC‑‑ACGACAGCAAGAAATTGACCGCGCTAAGGCCCGTAAAGAACGTCACGAGGGGCTCTTAGAGGCACGCAGATTCACACGTCGCAACCGCAAGGCACGTACAGCACCCAAAGCTAAGCGCGAAAGAATGCTTGCTGCGTGGCGATGGACTGAACGTCAAGa                                                                                                                                             >  1:161247‑M2/142‑301 (MQ=255)`  
                                                                                                                                                              `cctgtagttcgaacacttaggtctaatgcctaacaacatggacgtaatccgcatccggcaagcgcgtatatatgagtaattaacgattgacctctgggaaggcgcagaagactggctcaatgaatacttggagaaagtcgA‑‑CCGACAGAAAGAAATTGACCGCGCTAAGGCCCGTAAATAACGTCACGAGGGGCGCTTACAGGCACGCAGATTCAAACGTCGCAACAGCAAGGCACGTAAAGCACACAAAGCCAAGCGCGAAAGAATGCTTTCTGCGTGGGGATTGGCTGAACGTCAAGaa                                                                                                                                            <  1:330325‑M2/161‑1 (MQ=255)`  
                                                                                                                                                                             `gatgcctgacaccaaggacgtaatccgcatcctgcaagcgcgtatctatgagcaattaacgattgacctctgggaagacgcagaagacttgctcaatgaatacttggaggaagtcgaggaagtcgC‑‑ACGACAGAAAGAAATTGACCGCGCTAAGGCCCGTAAAGAACGTCACGAGGGGCGCTTAGAGGCACGCAGATTCAAACGTCGCAACCGCAAGGCACGTAAAGCACACAAAGCTAAGCGCGAAAGAATGCTTGCTGCGTGGCgatgggctgagcgtcaagctgtctcttatacacat                                                                                                                             >  1:219867‑M2/126‑284 (MQ=255)`  
                                                                                                                                                                             `ctctggtctgatgcctgacaccaaggacgtaatccgcatcctgcaagcgcgtatctatgagcaattaacgattgacctctgggaagacgcagaagacttgctcaatgaatacttggaggaagtcgC‑‑ACGACAGAAAGAAATTGACCGCGCTAAGGCCCGTAAAGAACGTCACGAGGGGCGCTTAGAGGCACGCAGATTCAAACGTCGCAACCGCAAGGCACGTAAAGCACACAAAGCTAAGCGCGAAAGAATGCTTGCTGCGTGGCGATGGGCTGAACGTCAAGAACGGCGTAACCATGAg                                                                                                                             <  1:307804‑M2/176‑1 (MQ=255)`  
                                                                                                                                                                               `ctggtctgatgcctgacaccaaggacgtaatccgcatcctgcaagcgcgtatctatgagcaattaacgattgacctctgggaagacgcagaagacttgctcaatgaatacttggaggaagtcgC‑‑ACGACAGAAAGAAATTGACCGCGCTAAGGCCCGTAAAGAACGTCACGAGGGGCGCTTAGAGGCACGCAGATTCAAACGTCGCAACCGCAAGGCACGTAAAGCACACAAAGCTAAGCGCGAAAGAATGCTTGCTGCGTGGCGATGGGCTGAACGTCAAGAACGGCGTAACCATGAGGt                                                                                                                           <  1:261344‑M2/178‑1 (MQ=255)`  
                                                                                                                                                                                    `ctgatgcctgacaccaaggacgtaatccgcatcctgcaagcgcgtatctatgagcaattaacgattgacctctgggaagacgcagaagactggctcaatgaatacttggaggaagtcgC‑‑ACGACAGAAAGAAATTGACCGCGCTAAGGCCCGTAAAGAACGTCACGAGGGGCGCTTAGAGGCACGCAGATTCAAACGTCGCAACCGCAAGGCACGTAAAGCACACAAAGCTAAGCGCGAAAGAATGCTTGCTGCGTGGCGATGGGCTGAACGTCAAGAACGGCGTAACCATGAGGTAGCTg                                                                                                                      <  1:115587‑M2/183‑1 (MQ=255)`  
                                                                                                                                                                                      `gatgcctgacaccaaggacgtaatccgcatcctgcaagcgcgtatctatgagcaattaacgattgacctctgggaagacgcagaagacttgctcaatgaatacttggaggaagtcgC‑‑ACGACAGAAAGAAATTGACCGCGCTAAGGCCCGTAAAGAACGTCACTAGGGGCGCTTAGAGGCACGCAGATTCAAACGTCGCAACCGCAAGGCACGTAAAGCACACAAAGCTAAGCGCGAAAGAATGCTTGCTGCGTGGCGATGGGCTTAACGTCAAGAACGGCGTAACCATGAGGTAGCTGTa                                                                                                                    >  1:33992‑M2/117‑301 (MQ=255)`  
                                                                                                                                                                                        `ggcctgacaccaaggacgtaatccgcatcatgcaagcgcgtatctatgagcaattaacgattgacctctgggaagacgcagaagacttgctcaatgaatacttggaggaagtcgC‑‑ACGACAGAAAGAAATTGACCGCGCTAAGGCCCGTAAAGAACGTCACGAGGGGCGCTTAGAGGCACGCAGATTCAAACGTCGCAACCGCAAGGCACGTAAAGCACACAAAGCTAAGCGCGAAAGAATGCTTGCTGCGTGGCGATGGGCTGAACGTCAAGAACGGCGTAACCACGAGGTAGCTGTAGa                                                                                                                  <  1:214200‑M2/187‑1 (MQ=255)`  
                                                                                                                                                                                         `gcctgacaccaaggacgtaatccgcatcctgcaagcgcgtatctatgagcaattaacgattgacctctgggaagacgcagaagacttgctcaatgaatacttggaggaagtcgC‑‑ACGACAGAAAGAAATTGACCGCGCTAAGGCCCGTAAAGAACGTCACGAGGGGCGCTTAGAGGCACGCAGATTCAAACGTCGCAACCGCAAGGCACGTAAAGCACACAAAGCTAAGCGCGAAAGAATGCTTGCTGCGTGGCGATGGGCTGAACGTCAAGAACGGCGTAACCATGAGGTAGCTGgagcg                                                                                                                 >  1:118569‑M2/114‑299 (MQ=255)`  
                                                                                                                                                                                         `gactgacaccaaggacgtaagccgcatcctgcaagcgcgtatctatgagcaattaacgattgacctctgagaagacgcagaagacttgctcaatgaatacttggaggaagtcgC‑‑ACGACAGAAAGAAATTGACCGCGCTAAGGCCCGTAAAGAACGTCACGAGGGGCGCTTAGAGGCACGCAGATTCAAACGTCGCAACCGCAAGGCACGTAAAGCACACAAAGCTAAGCGCGAAAGAATGCTTGCTGCGTGGCGATGGACTGAACGTCAAGAACGGCGTAACCATGAGGTAGCTGTAGAt                                                                                                                 <  1:3716‑M2/188‑1 (MQ=255)`  
                                                                                                                                                                                         `aaaggacgtaatccgcatcctgcaagcgcgtatctatgagcaattaacgattgacctctgggaagacgcagaagacttgctcaatgaatacttggaggaagtcgaggaagtcgC‑‑ACGACAGAAAGAAATTGACCGCGCTAAGGCCCGTAAAGAACGTCACGAGGGGCGCTTAGAGGCACGCAGATTCAAACGTCGCAACCGCAAGGCACGTAAAGCACACAAAGCTAAGCGCGAAAGAATGCTTGCTGCGTGGCGATGGGCTGAACGTCAAGAACGGCGTAACCATGAGGTAGCTGTAGAt                                                                                                                 <  1:22265‑M2/188‑1 (MQ=255)`  
                                                                                                                                                                                          `cctgacaccaaggacgtaatccgcatcctgcaagcgcgtatctatgagcaattaacgattgacctcttggaagacgcagaagacttgctcaatgaatacttggaggaagtcgC‑‑ACGACAGAAAGAAATTGACCGCGCTAAGGCCCGTAAAGAACGTCACGAGGGGCGCTTAGAGGCACGCAGATTCAAACGTCGCAACCGCAAGGCACGTAAAGCACACAAAGCTAAGCGTGAAAGAATGCTTGCTGCGTGGCGATGGGCTGAACGTCAAGAACGGCGTAACCATGAGGTAGCTGTAGATg                                                                                                                <  1:127456‑M2/189‑1 (MQ=255)`  
                                                                                                                                                                                            `ggacgtaatccgcatcctgcaagcgcgtatctatgagcaattaacgattgacctctgggaagacgcagaagacttgctcaatgaatacttggaggaagtcgaggaagtcgC‑‑ACGACAGAAAGAAATTGACCGCGCTAAGGCCCGTAAAGAACGTCACGAGGGGCGCTTAGAGGCACGCAGATTCAAACGTCGCAACAGCAAGGCACGTAAAGCACACAAAGCTAAGCGCGAAAGAATGCTTGCTGCGTGGCGATGGGCTGAACGTAAAGAACGGCGTAACCATGAGGTAGCTGTAGATgtt                                                                                                              >  1:240543‑M2/111‑300 (MQ=255)`  
                                                                                                                                                                                             `gacaccaaggacgtaatccgcatcctgcaagcgcgtatctatgagcaattaacgattgacctctgggaagacgcagaagacttgctcaatgaatacttggaggaagtcgC‑‑ACGACAGAAAGAAATTGACCGCGCTAAGGCCCGTAAAGAACGTCACGAGGGGCGCTTAGAGGCACGCAGATTAAAACGTCGCAACCGCAAGGCACGTAAAGCACACAAAGCTAAGCGCGAAAGAATGCTTGCTGCGTGGCGATGGGCTGAACGTCAAGAACGGCGTAACCAAGAGGTAGCTGTAGATGTAc                                                                                                             <  1:78424‑M2/192‑1 (MQ=255)`  
                                                                                                                                                                                             `aacaccaaggacgtaacccacatcctgcaagcgcgtatctatgagcaattaacgattgacctcttggaagacgcagaagacttgctcaatgaatacttggaggaagtagC‑‑ACGACAGAAAGAAATTGACCGTGCTAAGTCCCGTAAAGAACGTCACGAGGGGCGCTTAGAGGCACGCAGATTCAAACGTCGCAACCGCAAGGCACGTAAAGCACACAAAGCTAAGCGCGAAAGAATGCTTGCTGCGTGGCGATGGGCTGAACGTCAAGAACGGCGTAACCATGAGGTAGCTGTAGATGTAc                                                                                                             <  1:153381‑M2/192‑1 (MQ=255)`  
                                                                                                                                                                                               `aaggtaatccgcatcctgcaagcgcgtatctatgagaaattaacgattgacctctgggaagacgcagaagacttgctcaatgaatacttggaggaagtcgaggaagtCGAACGACAGAAAGAAATTGACCGCGCTAAGGCCCGTAAAGAACGTCACGAGGGGCGCTTAGAGGCACGCAGATTCAAACGTCGCAACCGCAAGGCACGTAAAGCACAAAAAGCTAAGCGCGAAAGAATGCTTGCTGCGTGGCGATGGGCTGAACGTCAAGAACGGCGTAACCATGAGGTAGCTGTAGATGTAc                                                                                                             <  1:252458‑M2/194‑1 (MQ=255)`  
                                                                                                                                                                                                  `caaggacgtaatccgcatcctgcaagcgcttatctatgagcaattaacgattgacctctgggaagacgcagaagacttgctcaatgaatacttggaggaagtcgC‑‑ACGACAGAAAGAAATTGACCGCGCTAAGGCCCGTAAAGAACGTCACGAGGGGCGCTTAGAGGCACGCAGATTCAAACGTCGCAACCGCAAGGCACGTAAAGCACACAAAGCTAAGCGCGAAAGAATGCTTGCTGCGTGGCGATGGGCTGAACGTCAAGAACGGCGTAACCATGAGGTAGCTGTAGATGTACTAGga                                                                                                        <  1:186845‑M2/197‑1 (MQ=255)`  
                                                                                                                                                                                                  `aatccgcatcctgcaagcgcgtatctatgagcaattaacgattgacctctgggaagacgcagaagacttgctcaatgaatacttggaggaagtcgaggaagtcgC‑‑ACGACAGAAAGAAATTGACCGCGCTAAGGCCCGTAAAGAACGTCATGAGGGGCGCTTAGAGGCACGCAGATTCAAACGTCGCAACCGCAAGGCACGTAAAGCAAACAAAGCTAAGAGCGAAAGAATGCTTGCTGCGGGGCGATGGGCTGAACGTcaagaacggcggaaccatgaggctggctctgaaacacaactc                                                                                                        >  1:54381‑M2/105‑281 (MQ=255)`  
                                                                                                                                                                                                  `aaaggacgtaagccgcatcctgcaagcgcgtatctatgagcaataaacgattgacctctgggaagacgcagaagacttgctcaatgaatacttggaggaagtcgC‑‑ACGACAGAAAGAAATTGACCGCGCGAAGGCCCGTAAAGAACGTCACGAGGGGCGCTTAGAGGCACGCAGATTCAAACGTCGCAACAGCAAGGCACGTAAAGCACACAAAGCTAAGCGCGAAAGAATGCTTGCTGCGTGGCGATGGGCTGAACGTCAAGAACGGCGTAACCATGAGGTAGCTGTAGATGTACTAGga                                                                                                        <  1:181238‑M2/197‑1 (MQ=255)`  
                                                                                                                                                                                                    `aggacgtaatccgcatcctgcaagcgcgtatctatgagcaattaacgattgacctctgggaagacgcagaagacttgctcaatgaatacttggaggaagtcgC‑‑ACGACAGAAAGAAATTGACCGCGCTAAGGCCCGTAAAGAACGTCACGAGGGGCGCTTAGAGGCACGCAGATTCAAACGTCGCAACCGCAAGGCACGTAAAGCACACAAAGCTAAGCGCGAAAGAATGCTTGCTGCGTGGCGATGGGCTGAACGTCAAGAACGGCGTAACCATTAGGTAGCTGTAGATGTACTAGgaag                                                                                                      <  1:21684‑M2/199‑1 (MQ=255)`  
                                                                                                                                                                                                      `gacgtaatccgcatcctgcaagcgcgtatctatgagcaattaacgattgacctctgggaagacgcagaagacttgctcaatgaatacttggaggaagtcgC‑‑ACGACAGAAAGAAATTGACCGCGCTAAGGCCCGTAAAGAACGTCACGAGGGGCGCTTAGAGGCACGCAGATTCAAACGTCGCAACCGCAAGGCACGTAAAGCACACAAAGCTAAGCGCGAAAGAATGCTTGCTGCGTGGCGATGGGCTGAACGTCAAGAACCGCGTAACCATGAGGTAGCTgtagatgtacctgtctctt                                                                                                    >  1:183774‑M2/101‑292 (MQ=255)`  
                                                                                                                                                                                                           `aatccgcatcctgcaagcgcgtatctatgagcaattaacgattgacctctgggaagacgcagaagacttgctcaatgaatacttggaggaagtcgC‑‑ACGACAGAAAGAAATTGACCGTGCTAAGGCCCGTAAAGAACGTCACGAGGGGCGCTTAGAGGCACGCAGATTCAAACGTCGCAACCGCAAGGCACGTAAAGCACACAAAGCTAAGCGCGAAAGAATGCTTGCTGCGTGGCGATGGGCTGAACGTCAAGAACGGCGTAACCATGAGGTAGCTGTAGATGTACTAGGAAGAACcaaa                                                                                               >  1:168654‑M2/96‑300 (MQ=255)`  
                                                                                                                                                                                                            `ctgcaagcgcgtatctatgagcaattaacgattgacctctgggaagacgcagaagacttgctcaatgaatacttggaggaagtcgaggaagtcgC‑‑ACGACAGAAAGAAATTGACCGCGCTAAGGCCCGTAAAGAACGTCACGAGGGGCTCTTAGAGGCACGCAGATTCAAACGTCGCAACCGCAAGGCACGTAAAGCACACAAAGCTAAGCGCGAAAGAATGCTTGCTGCGTGGCTATGGGCTGAACGTCAAGAACGGCGTAaccatgaggtagctgtagatctgtctcttatacacatct                                                                                              >  1:106341‑M2/95‑282 (MQ=255)`  
                                                                                                                                                                                                             `tccgcatcctgcaagcgcgtatctatgagcaattaacgattgacctctgggaagacgcagaagacttgctcaatgaatacttggagtaagtcgC‑‑ACGACAGAAAGAAATTGACCGCGCTAAGGCCCGTAAAGAACGTCACGAGGGGCGCTTAGAGGCACGCAGATTCAAACGTCGCAACCGCAAGGCACGTAAAGCACACAAAGCTAAGCGCGAAAGAATGCTTGCTGCGTGGCGATGGGCTGAACGTCAAGCACGGCGTCACCATGAGGTAGCTGTAGATGTACTAGGAAGAACCAATaa                                                                                             >  1:203549‑M2/94‑301 (MQ=255)`  
                                                                                                                                                                                                             `tccgcatcctgcaagcgcgtatctatgagcaattaacgattgacctctgggaagacgcagaagacttgctcaatgaatacttggaggaagtcgC‑‑ACGACAGAAAGAAATTGACCGCGCTAAGGCCCGTAAAGAACGTCACGAGGGGCGCTTAGAGGCACGCAGATTAAAACGTAGCAACCGCAAGGCACGTAAAGCACACAAAGCTAAGCGCGAAAGAATGCTTGCTGCGTGGCGATGGGCTGAACGTCAAGAACGGCGTAACCATGAGGTAGCTGTAGATGTACTAGGAAGAAACAATaa                                                                                             >  1:258195‑M2/94‑301 (MQ=255)`  
                                                                                                                                                                                                                `ccatcctgcaagcgcgtatctatgagcaattaacgattgacctctgggaagacgcagaagactggctcaatgaatacttggaggaagtcgC‑‑ACGACAGAAAGAAATTGACCGCGCTAAGGCCCGTAAAGAACGTCACGAGGGGCGCTTAGAGGCACGCAGATTCAAACGTCGCAACCGCAAGGCACGTAAAGCACACAAAGCTAAGCGCGAAAGAATGCTTGCTGCGTGGCGATGTGCTGAACGTCAAGAACGGCGTAACCATGAGGTAGCTGTAGATGTACTAGGAAGAACCAATAACGc                                                                                          <  1:228673‑M2/211‑1 (MQ=255)`  
                                                                                                                                                                                                                     `ctgcaagcgcgtatctatgagcaattaacgattgacctctgggaagacgcagaagacttgctcaatgaatacttggaggaagtcgC‑‑ACGACAGAAAGAAATTGACCGCGCTAAGGCCCGTAAAGAACGTCACGAGGGGCGCTTAGAGGCACGCAGATTCAAACGTCGCAACCGCAAGGCACGTAAAGCACACAAAGCTAAGCGCGAAAGAATGCTTGCTGCGTGGCGATGGGCTGAACGTCAAGAACGGCGTAACCATGAGGTAGCTGTAGATGTACTAGGAAGAACCAATAACgctactt                                                                                     >  1:175930‑M2/86‑298 (MQ=255)`  
                                                                                                                                                                                                                     `ctgcaagcgcgtatctatgagcaattaacgattgacctctgggaagacgcagaagacttgctcaatgaatacttggaggaagtcgC‑‑ACGACAGAAAGAAATTGACCGCGCTAAGGCCCGTAAAGAACGTCACGAGGGGCGCTTAGAGGCACGCAGATTCAAACGTCGCAACCGCAAGGCACGTAAAGCACACAAAGCTAAGCGCGAAAGAATGCTTGCTGCGTGGCGATGGGCTGAACGTCAAGAACGGCGTAACCATGAGGTAGCTGTAGATGTACTAGGAAGAACCAATAACGCTATGc                                                                                     >  1:158915‑M2/86‑301 (MQ=255)`  
                                                                                                                                                                                                                     `agaatctatgagaaattaacgattgacctgtgggaagacgcagaagacttgctaaatgaatacttgggtgaagtcaaggaagtcgA‑‑ACGACAGAAAGAAATTGACCGCGCTAAGGCCCGTAAAGAACGTCACGAGGGGCGCTTAGAGGCACGCAGATTCAAACGTCGCAACCGAAAGGCACGTAAAGCACACAAAGCTAAGCGCGAAAGAATGCTTGCTGCGTGGCGATGGGCTGAACGTCAAGAACGGCGTAACCATGAGGTAGCTGTAGATGTACTAGGAAGAACCAATAACGCTATGc                                                                                     <  1:333720‑M2/216‑1 (MQ=255)`  
                                                                                                                                                                                                                       `tatctatgggcaattaacgattgacctctgggaagacgcagaagacttgctcaatgaatacttggaggaagtcgaggaagtcgC‑‑ACGACAGAAAGAAATTGACCGCGCTAAGGCCCGTAAAGAACGTCACGAGGGGCGCTTAGAGGCACGCAGATTCAAACGTCGCAACCGCAAGGCACGTAAAGCACACAAAGCTAAGCGCGAAAGAATGCTTGCTGCGTGGCGATGGGCTGAACGTCAAGAACGGCGTAACCATGAGGTAGCTGTAGATGTACTAGGAAGAACCAATAACGCTATGctc                                                                                   <  1:192190‑M2/218‑1 (MQ=255)`  
                                                                                                                                                                                                                       `tatctatgagcaattaacgattgacctctgggaagtcgcagaagacttgctcaatgaatacttggaggaagtcgaggaagtcgC‑‑ACTACAGATAGAAATTGACCGCGCTAAGGCCCGTAAAGAACGTCACGAGGGGCGCTTAGAGGCACGCAGATTCAAACGTCGCAACCGCAAGGCACGTAAAGCACACAAAGCTAAGCGCGAAAGAATGCTTGCTGCGTGGCGATGGGCTGAACGTCAAGAACGGCGTAACCATGAGGTAGCTGTAGATGTACTAGGAAGAACCAATAACGCTATGctc                                                                                   <  1:226126‑M2/218‑1 (MQ=255)`  
                                                                                                                                                                                                                            `atgagcaattaatgattgaccgctgggaagacgcagaagacttgctcaatgaatacttggaggaaggcgaggaagtcgC‑‑ACGACAGAAAGAAATTGACCGCGCTAAGGCCCGTAAAGAACGTCACGAGGGGCGCTTAGAGGCACGCAGATTCAAACGTCACAACCGCAAGGCACGTAAAGCACACAAAGCTAAGCGCGAAAGAATGCTTGCTGCGTGGCGATGGGCTGAACGTCAAGAACGGCGTAACCATGAGGTAGCTGTAGATGTACTAGGAAGAACCAATAACGCTATGCTCTGGGt                                                                              <  1:121979‑M2/223‑1 (MQ=255)`  
                                                                                                                                                                                                                                 `atcgatgagcaattaacgattgacctctgggaagacgcagaagacttgctcaatgaatacttggaggaagtcgC‑‑ACGACAGAAAGAAATTGACCGCGCTAAGGCCCGTAAAGAACGTCACGAGGGGCGCTTAGAGGCACGCAGATTCAAACGTCGCAACCGCAAGGCACGTAAAGTACACAAAGCTAAGCGCGAAAGAATGCTTGCTGCGTGGCGATGGGCTGAACGTCAAGAACGGCGTAACCATGAGGTAGCTGTAGATGTACTAGGAAGAACCAATAACGCTATGCTCTGGGTCAACa                                                                         <  1:51090‑M2/228‑1 (MQ=255)`  
                                                                                                                                                                                                                                 `aaattacagattgactaatgggaagacgcaaaagacttgctcaatgagtacttggaggaagtcgaggaagtcgC‑‑ACGACAGAAAGAAAGTGACAGCGCTAAGGCCCGTAAAGAACGTCACGAGGGGAGCTTAGAGGAACGCAGATTCAAACGTCGCAACCGCAATGCACGTAAAGCACACAAAGCTAAGCGCGAAAGAATGCTTGCTGCGTGGCGATGAGCTGAACGTCAAGAACGGCGTAACCATGATGTAGCTGTAGATGTACTAAGAAGAACCAATAACGCTATGCTCTGGGTCAACa                                                                         <  1:134338‑M2/228‑1 (MQ=255)`  
                                                                                                                                                                                                                                  `tctatgagcaattaactattgacctctgggaagacgcagaagacttgctcaatgaatacttggaggaagtcgC‑‑ACGACAGAAAGAAATTGACCGCGCTAAGGCCCGTAAAGAACGTCACGAGGGGCGCTTAGAGGCACGCAGATTCAAACGTCGCAACCGCAAGGCACGTAAAGCACACAAAGCTAAGCGCGAAAGAATGCTTGCTGCGTGGCGATGGGCTGAACGTCAAGAACGGCGTAACCATGAGGTAGCTGTAGATGTACTAGGAAGAACCAATAACGCTATGCTCtgggtctgtct                                                                        >  1:141455‑M2/73‑296 (MQ=255)`  
                                                                                                                                                                                                                                  `tctatgagcaattaacgattgacctctgggaagacgcagaagacttgctcaatgaatacttggatgaagtcgC‑‑ACGACAGAAAGAAATTGACCGCGCTAAGGCCCGTAAAGAACGTCACGAGGGGCGCTTAGAGGCACGCAGATTCAAACGTCGCAACCGCAAGGCACGTAAAGCACACAAAGCTAAGCGCGAAAGAATGCTTGCTGCGTGTCGATGGGCTGAACGTCAAGAACGGCGTAACCATGATGTAGCTGTAGATGTACTAGGAAGAACCAATAACGCTATGCCCTGGGTccacac                                                                        >  1:172866‑M2/73‑300 (MQ=255)`  
                                                                                                                                                                                                                                  `tctatgagcaattaacgattgacctctgggaagacgcagaagacttgctcaatgaatacttggaggaagtcgC‑‑AAGACAGAAAGAAATTGACCGCGCTAAGGCCCGTAAAGAACGTCACGAGGGGCGCTTAGAGGCACGCAGATTCAAACGTCGCAACCGCAAGGCACGTAAAGCACACAAAGCTAAGCGCGAAAGAATGCTTGCTGCGTGGCGATGGGCTGAACGTCAAGAACGGCGTAACCATGAGGTAGCTGTAGATGTACTAGGAAGAACCAATAACGCTATGCTCTGGGTCAACAt                                                                        <  1:75818‑M2/229‑1 (MQ=255)`  
                                                                                                                                                                                                                                    `tatgagcaattaacgattgacctctgggaagacgcagaagacttgctcaatgaatacttggaggaagtcgC‑‑ACGACAGAAAGAAATTGACCGCGCTAAGGCCCGTAAAGAACGTCACGAGTGGCGCTTAGAGGCACGCAGATTCAAACGTCGCAACCGCAAGGCACGTAAAGCACACAAAGCTAAGCGCGAAAGAATGCTTGCTGCGTGGCGATGGGCTGAACGTCAAGAACGGCGTAACCATGAGGTAGCTGTAGATGTACTAGGAAGAACCAATAACGCTATGCTCTGGGTCAACATGt                                                                      <  1:728‑M2/231‑1 (MQ=255)`  
                                                                                                                                                                                                                                      `tgagcaattaacgattgacctctgggaagacgcagaagacttgctcaatgaatacttggaggaagtcgA‑‑ACGACAGAAAGAAATTGACCGCGCTAAGGCCAGTAAAGAACGTCACGAGGGGCGCTTAGAGGCACGCAGATTCAAACGTCGCAACCGCAAGGCACGTAAAGCACACAAAGCTAAGCGCGAAAGAATGCTTGCTGCGTGGCGATGGGCTGAACGTCAAGAACGGCGTAACCATGAGGTAGCTGTAGATGTACTAGGAAGAACCAATAACGCTATGCTCTGGGTCAACATGTtc                                                                    <  1:70707‑M2/233‑1 (MQ=255)`  
                                                                                                                                                                                                                                      `aacgattgacctctgggaagacgcagaagacttgctcaatgaatacttggaggaagtcgaggaagtcgC‑‑ACGACAGAAAGAAATTGACCGCGCTAAGGCCCGTAAAGAACGTCACGAGGGGCGCTTAGAGGCACGCAGATTCAAACGTCGCAACCTCAAGGCACGTAAAGCACACAAAGCTAAGCGCGAAAGAATGCTTGCTGCGTGGCGATGGGCTGAACGTCAAGAACGGCGTAACCATGAGGTAGCTGTAGATGTACTAGGAAGAACCAATAACGCTATGCTCTGGGTCAACATGTtc                                                                    <  1:237011‑M2/233‑1 (MQ=255)`  
                                                                                                                                                                                                                                      `aacgattgacctctgggaagacgcagaagacttgctcaatgaatacttggaggaagtcgaggaagtcgC‑‑ACGACAGAAAGAAATTGACCGCGCTAAGGCCCGTAAAGAACGTCACGAGGGGCGCTTAGAGGCACGCAGATTCAAACGTCGCAACCGCAAGGCACGTAAAGCACACAAAGCTAAGCGCGAAAGAATGCTTGCTGCGTGGCGATGGGCTGAACGTCAAGAACGGCGTAACCATGAGGTAGCTGTAGAAGTACTAGGAAGAACCAATAACGCTATGCTCTGGGTCAACATGTtc                                                                    <  1:42667‑M2/233‑1 (MQ=255)`  
                                                                                                                                                                                                                                        `cgattgacctctgtgaagacgcagaagacttgctcaatgaatacttggaggaagtcgaggaagtcgC‑‑ACGACAGATAGAAATTGACCGCGCTAAGGCCCGTAAAGAACGTCACGAGGGGCGCTTAGAGGCACGCAGATTCAAACGTCGCAACCGCAAGGCACGTAAAGCACACAAAGCTAAGCGCGAAAGAATGCTTGCTGCGTGGCGATGGGCTGAACGTCAAGAACGGCGTAACCATGAGGTAGCTGTAGATGTACTAGGAAGAACCAATAACGCTATGCTCTGGGTCAACATGTtctc                                                                  <  1:268541‑M2/235‑1 (MQ=255)`  
                                                                                                                                                                                                                                        `cgaaattaacgatttacttctgggaagacgcagaagacttgctcaatgaatacttggaggaagtcgC‑‑ACGACAGAAAGATATTGACCGCGCTAAGGCCCGTAAAGAACGTCACGAGGGGCGCTTAGAGGCACGCAGATTCAAACGTCGCAACCGCAAGGCACGTAAAGCACACAAAGCTAAGCGCGAAAGAATGCTTGCTGCGTGGCGATGGGCTGAACGTCAATAACTGCGTAACCATGAGGTAGCTGTAGATGTACTAGGAAGAACCAATAACGCTATGCTCTGGGTCAACATGTtctc                                                                  <  1:100236‑M2/235‑1 (MQ=255)`  
                                                                                                                                                                                                                                        `agcaattaacgattgacctcagggaagacgcagaaaacttgctcaaggaaaacttggagaaagtcgC‑‑ACGACAGAAAGAAATTGACCGCGCTAAGGCCCGTAAAGAACGTCACGAGGGGCGCTTAGAGGCACGCAGATTCAAACGTCGCAACCGCAAGGCACGTAAAGCACACAAAGCTAAGCGCGAAAGAATGCTTGCTGCGTGGCGATGGGCTGAACGTCAAGAACGGCGTAACCATGAGGTAGCTGTAGATGTACTAGGAAGAACCAATAACGCTATGCTCTGGGTCAACATGTtctc                                                                  <  1:234533‑M2/235‑1 (MQ=255)`  
                                                                                                                                                                                                                                        `aaagagacaggattgacctctgggaagacgcagaagacttgctcaatgaatacttggaggaagtcgC‑‑ACGACAGAAAGAAATTGACCGCGCTAAGGCCCGTAAAGAACGTCACGAGGGGCGCTTAGAGGCACGCAGATTCAAACGTCGCAACCGCAAGGCACGTAAAGCACACAAAGCTAAGCGCGAAAGAATGCTTGCTGCGTGGCGATGGGCTGAACGTCAAGAACGGCGTAACCATGAGGTAGCTGTAGATGTACTAGGAAGAACCAATAACGCTATGCTCTGGGTCAACATGTtctc                                                                  <  1:336979‑M2/235‑1 (MQ=255)`  
                                                                                                                                                                                                                                          `caattaacgattgacctctgggaagacgcagaagacttgctcaatgaatacttggaggaagtcgC‑‑ACGACAGAAAGAAATTGACCGCGCTAAGGCCCGTAAAGAACGTCACGAGGGGCGCTTAGAGGCACGCAGATTCAAACGTCGCAACCGCAAGGCACGTAAAGCACACAAAGCTAAGCGCGAAAGAATGCTTGCTGCGTGGCGATGGGCTGAACGTCAAGAACGGCGTAACCATGAGGTAGCTGTAGATGTACTAGGAAGAACCAATAACGCTATGCTCTGGGTCAACATGTTCTCTg                                                                >  1:137304‑M2/65‑301 (MQ=255)`  
                                                                                                                                                                                                                                            `attaactattgacctctgggaagacgcagaagacttgctcaatgaatacttggaggaagtcgC‑‑ACGACAGAAAGAAATTGACCGCGCTAAGGCCCGTAAAGAACGTCACGAGGGGCGCTTAGAGGCACGCAGATTCAAACGTCGCAACCGCAAGGCACGTAAAGCACACAAAGCTAAGCGCGAAAGAATGCTTGCTGCGTGGCGATGGGCTGAACGTCAAGAACGGCGTAACCATGAGGTAGCTGTAGATGTACTAGGAAGAACCAATAAcgctatgctctgggtctgtctcttatacaca                                                              >  1:316065‑M2/63‑286 (MQ=255)`  
                                                                                                                                                                                                                                             `ataaggattggcgtctgggaggacggagaagacttacaagatgaataatggggggaagtcgA‑‑ACGACAGAAAGAAATTGACCGAGCAAAGGCCGGTAAAGAAGGACAAGAGGGGCGGTTAGAGGCAAGCAGAGTCAAACGTCGCAACCGAAAGGCACGTAAAGCACACAAAGCTAAGCGCGAAAGAATGCTTGCTGCGTGGCGATGGGCTGAACGTCAAGAACGGCGTAAACATGAGGTAGCTGTAGATGTACTAGGAAGAACCAATAACGCTATGCTCTGGGTCAACATGTTCTCTgggg                                                             <  1:211475‑M2/240‑1 (MQ=255)`  
                                                                                                                                                                                                                                                      `gacctctgggaagacgcagaagacttgctcaatgaatacttggaggaagtcgC‑‑ACGACAGAAAGAAATTGACCGCGCTAAGGCCCGTAAAGAACGTCACGAGGGGCGCTTAGAGGCACGCAGATTCAAACGTCGCAACCGCAAGGCACGTAAAGCACACAAAGCTAAGCGCGAAAGAATGCTTGCTGCGTGGCGATGGGCTGAACGTCAAGAACGGCGTAACCATGAGGTAGCTGTAGATGTACTAGGAAGAACCAATAACGCTATGCTCTGGGTAGACATGTTCTCTGGGGACTTTAAgg                                                    <  1:149565‑M2/249‑1 (MQ=255)`  
                                                                                                                                                                                                                                                      `gaccgctgggaagacgcagaagacttgctcaatgaatacttggaggaagtcgG‑‑ACGACAGAAAGAAATTGACCGGGCTAAGGCCCGTAAAGAACGTCACGAGGGGCGCTTAGAGGCACGCAGATTCAAACGTGGCAACCGCAAGGGACGTAAAGCACACAAAGCTAAGCGCGAAAGAATGCGTGCTGGGTGGCGATGGGCTGAACGTCAAGAACGGCGTAACCATGAGGTAGCTGTAGATGTACTAGGAAGAACCAAAAACGCTATGCTCTGGGTCAACATGTTCTCTGGGGACGTTAAgg                                                    <  1:207709‑M2/249‑1 (MQ=255)`  
                                                                                                                                                                                                                                                      `gaagacgcagaagacttgctcaatgaatacttggaggaaggcgaggaagtcgC‑‑ACGACAGAAAGAAATTTACCGCGCTAAGGCCCGTAAAGAACATCACGAGGGGCGCTTAGAGGCACGCAGATTCAAACGTCGCAACCGCAAGGCACATAAAGCACACAAAGCTAAGCGCGAAAGAATGCTTGCTGCGTGGCGATGGGCTGAACGTCAAGAACGGCGTAACCATGAGGTAGCTGTAGATGTACTAGGAAGAACCAATAACGCTATGCTCTGGGTCAACATGTTCTCTGGGGACTTTAAgg                                                    <  1:57260‑M2/249‑1 (MQ=255)`  
                                                                                                                                                                                                                                                           `ctgggaagacgcagaagacttgctcaatgaatacttggaggaagtcgC‑‑ACGACAGAAAGAAATTGACCGCGCTAAGGCCCGTAAAGAACGTCACGAGGGGCGCTTAGAGGCACGCAGATTCAAACGTCGCAACCGCAAGGCACGTAAAGCACACAAAGCTAAGCGCGAAAGAATGCTTGCTGCGTGGCGATGGGCTGAACGTCAAGAACGGCGTAACCATGAGGTAGCTGTAGATGTACTAGGAAGAACCAATAACGCTATGCTCTGGGTCAACATGTTCTCTGGGGACTTTAAGGCGCtt                                               >  1:277794‑M2/48‑301 (MQ=255)`  
                                                                                                                                                                                                                                                                   `acgcagaagacttgctcaatgaatacttggaggaagtcgC‑‑ACGACAGAAAGAAATTGACCGCGCTAAGACCCGTAAAGAACCTCACGAGGGGCTCTTATAGGCACGCAGATTCAAACGTCCCAACCGCAAGGCACGTAAAGCACACAAAGCTAAGCGCGAACTAATTCTTTCTGCGTGGCCCTGGGCTGACCGTCAAGCCCGGCGTAACCCTGAGGTCGCTGCATATGTACTAGTCATCACCACTCCCGCTCTGCCCTGTTTCAACATTTTCTCTTGGTCCTTTCAGGCGCTTTAGGAACg                                       >  1:131197/30‑301 (MQ=255)`  
                                                                                                                                                                                                                                                                      `aagaagacttgctcaatgaagacttggaggaagtcgC‑‑ACGACAGAAAGAAAGTGACCGCGCTAAGGCCCGTAAAGAACGTCACGAGGGGAGCTTAGAGGCACGCAGATGCAAACGTCGCAACCGAAAGGCACGTAAAGCACACAAAGCTAAGCGCGAAAGAATGCTTGCTGCGTGGCGATGGGCTGAACGTCAAGAACGGCGTAACCATGAGGTAGCTGTAGATGTACTAGGAAGAACCAATAACGCTATGCTCTGGGTCAACATGTTCTCTGGGGACTTTAAGGCGCTTGAGGAACGAAt                                    <  1:8240‑M2/265‑1 (MQ=255)`  
                                                                                                                                                                                                                                                                                       `gaatacttggaggaagtcgC‑‑ACGACAGAAAGAAATTGACCGCGCTAAGGCCCGTAAAGAACGTCACGAGGGGCGCTTAGAGGCACGCAGATTCAAACGTCGCAACCGCAAGGCACGTAAAGCACACAAAGCTAAGCGCGAAAGAATGCTTGCTGCGTGGCGATGGGCTGAACGTCAAGAACGTCGTAACCATGAGGTAGCTGTAGATGTACTAGGAAGAACCAATAACGCCATGCTCTGGGTCAACATGTTCTCTGGGGACCTTAAGGCGCTTGCGGAACGAATCGcgttgcacctgtccc                   >  1:313350‑M2/20‑294 (MQ=255)`  
                                                                                                                                                                                                                                                                                          `tacttggaggaagtcgC‑‑ACGACAGAAAGAAATTGACCGCGCTAAGGCCCGTAAAGAACGTCACGAGGGGCGCTTAGAGGCACGCAGATTCAAACGTCGCAACCGCAAGGCACGTAAAGCACACAAAGCTAAGCGCGAAAGAATGCTTGCTGCATGGCGATGGGCTGAACGTCAAGAACGGCGTAACCATGAGGTAGCTGTAGATGTACTAGGAAGAACCAATAACGCTATGCTCTGGGTCAACATGTTCTCTGGGGACTTTAAGGCGCTTGAGGAACGAAtcgcgctgcacctgtctctta                >  1:178658‑M2/17‑291 (MQ=255)`  
                                                                                                                                                                                                                                                                                           `acttggaggaagtcgC‑‑ACGACAGAAAGAAATTGACCGCGCTAAGGCCCGTAAAGAACGTCACGAGGGGCGCTTAGAGGCACGCAGATTCAAACGTCGCAACCGCAAGGCACGTAAAGCACACAAAGCTATGCGCGAAAGAATGCTTGCTGCGTGGCGATGGGCTGAACGTCAAGAACGGCGTAACCATGAGGTAGCTGTAGATGTACTAGGAAGAACCAATAACGCTATGCTCTGGGTCAACATGTTCTCTGGGGACTTTAAGGCGCTTGAGGAACGAATCGCGCTGCACTGGCGTAATGc               <  1:31891‑M2/286‑1 (MQ=255)`  
                                                                                                                                                                                                                                                                                           `aagtcgaggaagtcgC‑‑ACGACAGAAAGAAATTGACCGCGCTAAGGCCCGTAAAGAACGTCACGAGGGGCGCTTAGAGGCACGCAGATTCAAACGTCGCAACCGCAAGGCACGTAAAGCACACAAAGCTAAGCGCGAAAGAATGCTTGCTGCGTGGCGATGGGCTGAACGTCAAGAACGGCGTAACCATGAGGTAGCTGTAGATGTACTAGGAAGAACCAATAACGCTATGCTCTGGGTCAACATGTTCTCTGTGGACTTTAAGGCGCTTGAGGAACGAATCGCGCTGCACTGGCGTAATGc               <  1:298673/301‑1 (MQ=255)`  
                                                                                                                                                                                                                                                                                             `gtcgaggaagtcgC‑‑ACGACAGATAGAAATTGACCGCGCTAAGGCCCGTAAAGAACGTCACGAGGGGCGCTTAGAGGCACGCAGATTCAAACGTCGCAACCGCAAGGCACGTAAAGCACACAAAGCTAAGCGCGAAAGAATGCTTGCTGCGTGGCGATGGGCTGAACGTCAAGAACGGCGTAACCATGAGGTAGCTGTAGATGTACTAGGAAGAACCTATAACGCTATGCTCTGGGTCAACATGTTCTCTGGGGACTTTAAGGCGCTTGAGGAACGAATCGCGCTGCACTGGCGTAATGCTg             >  1:67676/1‑301 (MQ=255)`  

                                                                                                                                                                                                                                                                                                          `|`                                                                                                                                                                                                                                                                                                              
`GAGCGTTTCAACAATGATGCCCATTATAAATACGCTGAGATTGCAAGCGACATCATTGATTGCAATTCGGATGAGCATGATGAGTTAACTGGATGGGATGGTGAGTTTGTTGAAACTTGTAAACTAATCCGCAAGTTCTTTGAGGGCATCGCCTCATTCGACATGCATAGCGGGAACATCATGTTCTCAAATGGAGACGTACCATACATCACCGACCCGGTATCATTCTCGCAGAAGAAAGACGGTGGCGCATTCAGCATCGACCCTGAGGAACTCATCAAGGAAGTCGAGGAAGTCGC‑‑ACGACAGAAAGAAATTGACCGCGCTAAGGCCCGTAAAGAACGTCACGAGGGGCGCTTAGAGGCACGCAGATTCAAACGTCGCAACCGCAAGGCACGTAAAGCACACAAAGCTAAGCGCGAAAGAATGCTTGCTGCGTGGCGATGGGCTGAACGTCAAGAACGGCGTAACCATGAGGTAGCTGTAGATGTACTAGGAAGAACCAATAACGCTATGCTCTGGGTCAACATGTTCTCTGGGGACTTTAAGGCGCTTGAGGAACGAATCGCGCTGCACTGGCGTAATGCTGACCGGATGGCT  >  NC_001604.1/2444‑3040`

  

Alignment Legend

Aligned base mismatch/match (shaded by quality score): `ATCG/ATCG` `< 3 ≤` `ATCG/ATCG` `< 10 ≤` `ATCG/ATCG` `< 23 ≤` `ATCG/ATCG` `< 35 ≤` `ATCG/ATCG` `< 38 ≤` `ATCG/ATCG`

Unaligned base: `atcg`    Masked matching base: `atcg`    Alignment gap: `‑`    Deleted base: `‑`
